# Pulp Callus Extract From *Malus domestica* var. Mela Rosa Marchigiana Preserves Intestinal Barrier Integrity and Confers Neuroprotection in a Gut–Brain Axis Co‐Culture Model

**DOI:** 10.1002/biof.70139

**Published:** 2026-07-31

**Authors:** Alessandro Nicois, Daniele Fraternale, Francesco Palma, Brenda Marfella, Giuditta Fiorella Schiavano, Mariastella Colomba, Armando Gregorini, Barbara Di Giacomo, Luigia Cristino, Vincenzo Di Marzo, Maria Cristina Albertini, Letizia Palomba

**Affiliations:** ^1^ Department of Biomolecular Sciences University of Urbino Carlo Bo Urbino Italy; ^2^ Institute of Biomolecular Chemistry National Research Council of Italy Naples Italy; ^3^ Department of Biology University of Naples Federico II Naples Italy; ^4^ Department of Humanities University of Urbino Carlo Bo Urbino Italy; ^5^ Canada Excellence Research Chair on the Microbiome‐Endocannabinoidome Axis in Metabolic Health, Department of Medicine and School of Nutrition Université Laval Québec City Québec Canada; ^6^ Institut Universitaire de Cardiologie et de Pneumologie de Québec Université Laval Québec City Québec Canada; ^7^ Centre Nutrition, santé et société (NUTRISS), INAF, École de Nutrition Université Laval Québec City Québec Canada; ^8^ Joint International Research Unit Between Université Laval and National Research Council on Chemical and Biomolecular Studies on the Microbiome and Its Impact on Metabolic Health and Nutrition (JIRU‐MicroMeNu) Université Laval Québec City Québec Canada

**Keywords:** apoptosis, bioactive compounds, inflammation, oxidative stress, phytochemicals

## Abstract

The gut–brain axis is a key target in neuroinflammatory disorders. We investigated the protective effects of Mela Rosa Marchigiana pulp callus extract (MRME), a phytocomplex with a unique triterpenic profile. Using a validated transwell co‐culture model of the intestinal‐neural interface, differentiated Caco‐2 cells formed a polarized epithelial barrier (apical), while BV2 microglia or SH‐SY5Y neurons were seeded in the basolateral compartment. Apical MRME pretreatment preserved Caco‐2 barrier integrity against lipopolysaccharide or dextran sodium sulfate‐induced damage. MRME maintained occludin integrity and transepithelial electrical resistance (TEER), effectively neutralizing “leaky gut”‐like conditions. By stabilizing the barrier, MRME exerted indirect neuroprotection since high‐throughput live‐cell imaging revealed dose‐dependent reductions in reactive oxygen species generation and apoptosis (caspase‐3/7 activation) in both BV2 and SH‐SY5Y cells. MRME demonstrated a microbiologically neutral profile, exerting no inhibitory effects on either pathogenic or probiotic strains up to 10,000 μg/mL. MRME provides dual protection by strengthening the intestinal barrier and shielding the neural environment from systemic inflammation through host cellular modulation rather than microbial interference. These findings suggest MRME as a promising nutraceutical candidate for gut–brain axis dysregulation.

## Graphical abstract

Experimental design of *Malus x domestica* callus extract (MRME). Bioactive‐rich extract was obtained from callus culture derived from MRM apple. In a co‐culture model of Caco‐2, BV2, and SH‐SY5Y cells, LPS and DSS led to an increased production of reactive oxygen species (ROS) and apoptosis that was prevented by MRME. These findings suggest that MRME, preserving the integrity of the gut–brain axis and maintaining a balanced gut microbiome, promotes indirect neuroprotection.

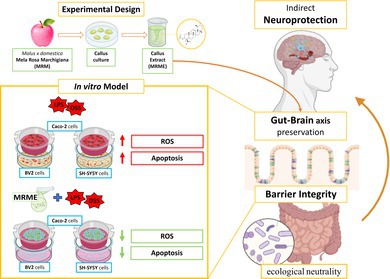

**Abbreviations**
DSS: dextran sodium sulfate
LPS: lipopolysaccharide
MRME: Mela Rosa Marchigiana pulp callus extract
ROS: reactive oxygen species
TEER: transepithelial electrical resistance

## 1 Introduction

The gut–brain axis is recognized as a complex, bidirectional communication network that plays a fundamental role in maintaining systemic homeostasis and modulating neurological health [1, 2, 3]. At the core of this interaction lies the intestinal epithelial barrier, a specialized physical and functional interface that regulates the passage of nutrients while preventing the translocation of pathogens and proinflammatory molecules [4, 5]. The integrity of this barrier is primarily maintained by tight junction (TJ) proteins, such as occludin, claudins, and zonula occludens‐1 which seal the paracellular space [6, 7]. Emerging evidence indicates that disruption of this barrier, often referred to as “leaky gut,” represents not merely a localized intestinal dysfunction but a key driver of systemic inflammation and neuroinflammatory processes, potentially contributing to neurodegenerative disease onset, including Alzheimer's and Parkinson's diseases [8, 9, 10, 11].

Lipopolysaccharide (LPS) and dextran sodium sulfate (DSS) are frequently used as stressors to induce intestinal damage in experimental models [12]. LPS, a constituent of Gram‐negative bacteria, triggers oxidative stress and inflammatory cascades through the activation of Toll‐like receptors 4 [13, 14]. Similarly, DSS is a chemical colitogen that disrupts the epithelial lining and increases permeability [12]. Both stimuli promote excessive production of reactive oxygen species (ROS) and activation of apoptotic pathways, as evidenced by caspase‐3/7 cleavage [15, 16, 17, 18]. In the context of the gut–brain axis, these intestinal insults can transmit distress signals to the central nervous system via vagal, immune, and metabolic pathways, thus activating resident immune cells like microglia and impairing neuronal viability [19, 20]. To counteract these harmful effects, there is growing interest in natural bioactive compounds derived from the agrobiodiversity [21]. The Mela Rosa Marchigiana (MRM) is an ancient apple cultivar native to the Marche region, Italy, renowned for its resilience and unique phytochemical profile [22]. The present study used an ethanolic extract of callus obtained from the in vitro culture of this apple pulp (MRME), characterized by a high concentration of pentacyclic triterpenic acids [23]. MRME exerts antioxidant activity as evidenced by DPPH (67%) and ABTS (39%) assays [24]. Beyond its chemical scavenging ability, this extract mitigates ROS production in keratinocytes and confers genoprotection as observed in a nicking assay [24]. Furthermore, MRME reduces proinflammatory mediators such as IL‐1β, IL‐8, and MCP‐1 induced by LPS, and modulates miRNAs linked to inflammation and senescence [25]. MRME pulp‐derived callus cultures offer a stable production of bioactive triterpenes, maintaining anti‐inflammatory activity in all models tested [23], confirming its potential as a sustainable source of functional compounds. The MRME unique phytochemical profile, characterized by high concentration of pentacyclic triterpenes, provides a strong premise for further exploring its bioactivity within the gut–brain axis.

Indeed, these compounds, particularly tormentic and annurcoic acids, have been shown to reinforce epithelial junctions and stabilize the mucosal barrier [26, 27], suggesting that MRME may act as a strategic gatekeeper against the systemic spread of neuroinflammatory triggers. Despite the ancient origin of this cultivar and the recognized health benefits associated with its consumption, the molecular mechanisms underlying MRME‐mediated protection within the gut–brain axis remain largely unexplored. In this study, we employed a well‐established and validated co‐culture model to simulate this communication. Differentiated Caco‐2 cells, a widely used human intestinal epithelial cell line mimicking the intestinal epithelium, were grown on transwell inserts to form a functional barrier [28, 29]. In the basolateral compartment, we positioned either BV2 microglial cells or SH‐SY5Y neurons to monitor the effects of intestinal stress on a “brain‐like” environment.

The primary objective of this study was to determine whether apically administered MRME preserves intestinal barrier integrity and indirectly prevents ROS generation and apoptosis in the underlying microglial or neuronal cell layers. Furthermore, to ensure the safety and potential prebiotic applicability of the extract, we assessed its impact on the proliferation and viability of both Gram‐positive and Gram‐negative bacterial strains, aiming to confirm a neutral or beneficial profile toward the gut microbiota [30, 31].

Our findings suggest that MRME, by reinforcing the intestinal barrier, indirectly protects neural cells from oxidative stress and apoptosis, offering a potential nutraceutical approach for maintaining gut–brain homeostasis.

## 2 Material and Methods

### 2.1 Callus Culture and Mela Rosa Marchigiana Ethanolic Extract (MRME) Preparation

The callus obtained from the pulp fruit of Mela Rosa Marchigiana (*Malus x domestica* Borkh var. Mela Rosa Marchigiana MRM) used in this study and its ethanolic extract (MRME) are the same as those used in Gubitosa et al. [23]. Specifically, the callus is derived from long‐term stabilized subcultures of the material originally characterized by Verardo et al. [32]. Briefly, we employed the Gamborg B5 medium plus 2.0 mg/L 6‐benzylaminopurine (BA, Sigma‐Aldrich) and 0.2 mg/L 2,4‐D (2,4‐dichlorophenoxyacetic acid, Sigma‐Aldrich), plus 30 g/L sucrose, pH 5.8, as this combination turned out to give the highest biomass production from pulp explants of MRM. Callus culture was conducted in the dark at 25°C ± 2°C. Subcultures into fresh medium were performed every 28 days to keep the cellular material alive. The remainder was collected and stored at −20°C until use. When necessary, part of the frozen material was freeze‐dried and used for the extraction procedure.

### 2.2 Cell Cultures and Treatments

The SH‐SY5Y human neuroblastoma cell line (CRL‐2266; American Type Culture Collection, Manassas, VA, USA) was cultured in Dulbecco's Modified Eagle's Medium (DMEM) supplemented with 20% fetal bovine serum (FBS), 1% antibiotic–antimycotic mixture, and 2 mmol/L L‐glutamine [33]. BV2 cells (CellTec Systems GmbH, Osterweide 2c, Lübeck, Germany), immortalized murine microglial cells derived from C57/BL6 mice, were cultured in RPMI medium supplemented with 10% FBS and 1% Penicillin–Streptomycin [34]. Caco‐2 cells (Creative Biogenes, Shirley, New York 11967, United States), derived from human colon carcinoma, were cultured in high‐glucose DMEM supplemented with 10% FBS, 1% Penicillin–Streptomycin, 1% Essential Amino Acids, and 20 mM HEPES. All cell lines were maintained in a humidified incubator at 37°C with 5% CO_2_, and all reagents were from GIBCO (Invitrogen, Carlsbad, CA, USA). Cells were split and plated after reaching 80% confluency, with densities ranging from 4500 to 6000 cells/cm^2^ at each passage.

To allow cell polarization and differentiation into intestinal epithelial cells, Caco‐2 cells were seeded at a density of 65,000 cells per well onto 12‐well PET tissue culture inserts (growth area: 1.1 cm^2^; pore size: 0.4 μm; Merck, Darmstadt, Germany) and cultured for 21 days. The formation of a continuous epithelial barrier was confirmed by measuring transepithelial electrical resistance (TEER) [28, 29].

Differentiated Caco‐2 cells were treated with increased (1, 10, and 100 μg/mL) MRME concentrations in serum free medium. After 24 h, the medium was replaced with new culture medium containing 0.1 μg/mL LPS or 1% DSS. After a 12‐h treatment, Caco‐2 cells were fixed with 4% PFA for subsequent immunofluorescence assays.

### 2.3 Trans‐Epithelial Electrical Resistance (TEER)

TEER is a noninvasive, label‐free method used to measure the electrical resistance across a cellular monolayer, which indicates the integrity and tightness of intercellular junctions [29, 35]. TEER measurements were determined using a millicell ERS meter (Millipore Corporation). The instrument consists of a pair of chopstick electrodes that are placed on both sides of a cellular monolayer grown on a permeable membrane [28]. A small, noninvasive alternating current voltage is applied to calculate the barrier's resistance. This measurement quantifies the ionic conductance across the TJs between cells [4]. Caco‐2 cells were monitored at specific time intervals before and after treatments. TEER was recorded at each time point. The values were expressed as ohmic resistance values and normalized to the control well [29].

### 2.4 Immunofluorescence

Caco‐2 fixed cells were permeabilized for 10 min with phosphate buffer (PB) containing 0.3% Triton X‐100 (Sigma‐Aldrich, St. Louis, MO, USA) and subsequently incubated overnight with the primary antibody antioccludin (1:500, mouse monoclonal CL1555, AbCam, Cambridge, UK) [6]. After washing, the samples were incubated for 2 h with a mixture of the appropriate secondary antibody, AlexaFluor Donkey antimouse 594 (1:500), and the nuclear stain DAPI (1 mg/mL; Sigma‐Aldrich) [36]. Finally, the cells were mounted with an aqueous mounting medium (Aquatex, Merck, Darmstadt, Germany). Fluorescence‐labeled cells were analyzed with a Leica DMI6000 fluorescence microscope equipped with an *x*–*y*–*z* motorized stage and a cooled digital camera (Leica K5; Leica Microsystems, Buccinasco, Milan, Italy). Images were digitally acquired at the same magnification and processed for fluorescence quantification using Leica LAS X software (Leica Application Suite X 3.7.4.23463). The optimal focus was chosen for each fluorescence channel from the *z*‐stacked images, which were then merged to obtain the best multichannel image for each analyzed well to measure the intensity of the fluorescent signal for each immunostaining [37]. All measurements were performed in quadruplicate. Statistical and quantification analyses were conducted by an observer blinded to the experimental design using Fiji, an image‐processing package of ImageJ software (version 2.14.0) developed by the National Institutes of Health, USA [37].

### 2.5 ROS Measurement in Co‐Culture Model

To evaluate the induction of oxidative stress in the basolateral compartment of the co‐culture, the differentiated Caco‐2 cells seeded on the transwell inserts were treated as detailed above. Following Caco‐2 treatment, BV2 and SH‐SY5Y cells seeded in the lower chamber were incubated with 2.5 μM CellRox reagent diluted in a serum‐free medium for 30 min at 37°C. To capture the dynamic production of ROS, the plates were placed into the IncuCyte live‐cell scanner chamber (Sartorius, Gottingen, Germany) and automated phase contrast and fluorescence images were acquired. The integrated IncuCyte software was used to quantify the fluorescence intensity. Mean fluorescence values were determined by averaging the fluorescence values of at least 50 cells/treatment condition/experiment.

### 2.6 Caspase‐3/7 Measurement in Co‐Culture Model

To evaluate apoptosis in BV2 and SH‐SY5Y cells seeded in the lower chamber of the co‐culture, differentiated Caco‐2 cells were treated with LPS or DSS in the absence or presence of MRME (see Section 2.2). After treatments, BV2 and SH‐SY5Y cells were incubated with the IncuCyte caspase‐3/7 green apoptosis assay reagent (Sartorius, Gottingen, Germany 1:1000) in serum‐free medium and placed in the IncuCyte Live Cell analysis system at 37°C. Images were automatically acquired at regular intervals throughout the experiment using phase contrast and green fluorescence channels. The integrated IncuCyte software was used to quantify the fluorescence intensity of at least 50 cells/treatment condition/experiment.

### 2.7 Bacterial Strains

The effect of MRME on bacterial growth was evaluated in vitro at different extract concentrations (10,000, 200, 100, 10, and 1 μg/mL) using both pathogen and probiotic gut strains. As gut pathogen bacteria, the tested strains were *Salmonella enteritidis* ATCC 13076 and *Enterococcus faecalis* ATCC 29212, which were grown on Tryptic Soy Agar (TSA) and Slanetz plates, respectively. Moreover, three probiotic strains were used: *Lactococcus lactis* subsp. *lactis* NCTC 6681, *Lactobacillus fermentum* ATCC 9338, and *Lactobacillus delbrueckii* subsp. *bulgaricus* NCTC 12712, and the growth probiotic agar was M17 and Man Rogosa Sharpe (MRS) (Liofilchem, Roseto degli Abruzzi, Italy), respectively.

### 2.8 Microorganism Suspensions Preparation

*S. enteritidis* and *E. faecalis* were grown overnight in Muller Hinton Broth (MHB) at 35°C for 18–24 h in aerobic conditions. While *L. lactis* , *L. fermentum* , and *L. d. bulgaricus* were grown overnight in MRS broth +1 mL/L Tween 80 at 35°C for 24–48 h in anaerobic conditions (GasPak EZ Anaerobe Pouch System with Indicator, BD GasPak EZ chamber). The bacterial suspensions were standardized to 0.5 McFarland scale (10^8^ CFU/mL) using spectrophotometric measurement, setting an optical density of 600 nm (OD_600_), and then diluted to obtain a bacterial suspension of 10^6^ CFU/mL. All bacterial suspension concentrations were experimentally confirmed by performing 10‐fold serial dilutions in saline solution (NaCl 0.9%, w/v). Ten microliters of each dilution suspension were spread‐plated in triplicate on appropriate agar and incubated at 35°C for 24–48 h in appropriate conditions for the enumeration of CFU/mL.

### 2.9 Antimicrobial MRME Activity Assay

To evaluate the MRME antimicrobial activity, 5 mL (10^6^ CFU/mL) of each bacterial suspension was added with different volumes of MRME to reach a final MRME concentration of 10,000, 200, 100, 10, and 1 μg/mL. As positive controls, only bacterial suspension (CTR) and bacterial suspension added with MRME vehicle (70% EtOH in sterile water) (VEH) were considered. After incubation at 35°C for 24–48 h, the log_10_ CFU/mL was calculated on the appropriate agar plates.

### 2.10 Statistical Analyses

Statistical analysis was performed using GraphPad Prism 10 [38]. All grouped datasets were evaluated using a one‐ or two‐way ANOVA test with a Sidak or Bonferroni post hoc test. A *p* ≤ 0.05 was considered statistically significant. All data underwent descriptive statistics and tests for lognormality (Kolmogorov–Smirnov or Shapiro–Wilk) to define the data distribution [39].

## 3 Results

### 3.1 MRME Preserves Intestinal Epithelial Barrier Integrity

MRME is a complex mixture of pentacyclic triterpenic acids and phytosterols predominantly characterized by tormentic acid (40.2%), followed by annurcoic acid (16.6%), maslinic acid (12.2%), corosolic acid (11.8%), and ursolic acid (10.6%), with minor amounts of oleanolic acid, pomolic acid, and β‐sitosterol (data from GC/MS and GC FID analyses, see Gubitosa et al. [23]).

**FIGURE 1:**
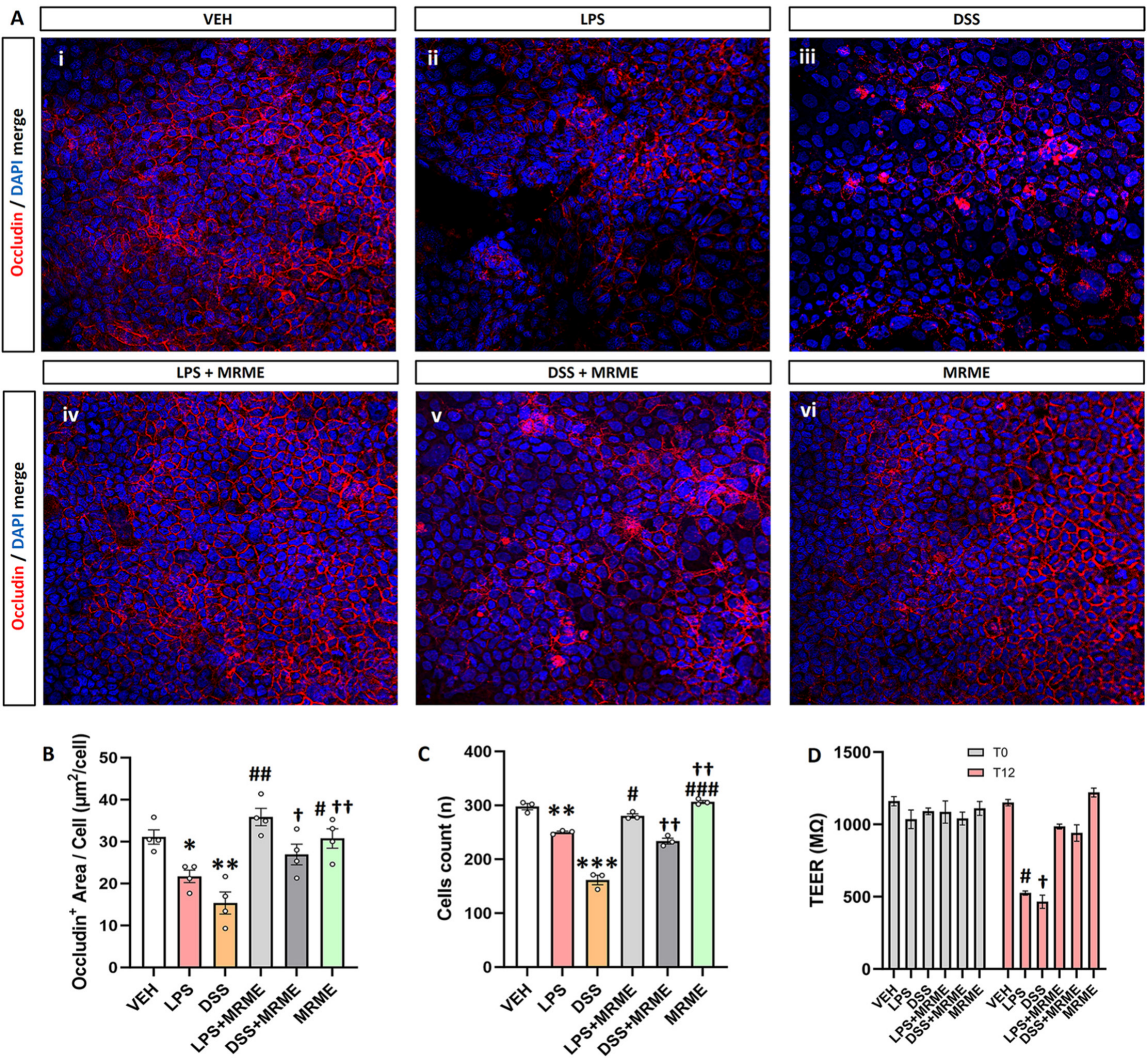
MRME preserves intestinal barrier integrity. Representative micrographs of occludin (red) immunocytochemical and DAPI (blue) staining in differentiated Caco‐2 enterocytes under the following conditions: (i) vehicle (VEH), (ii) LPS (0.1 μg/mL, 12 h), (iii) DSS (1%, 12 h), (iv) MRME + LPS, (v) MRME + DSS (1%), and (vi) MRME alone (10 μg/mL, 24 h) (A). The cells were preincubated with MRME (10 μg/mL, 24 h) prior to exposure to LPS (0.1 μg/mL) or DSS (1%) for 12 h. (B) Occludin fluorescence intensity. (C) Caco‐2 cell count. (D) TEER measurements with the baseline value (*t* = 0) in gray and pink bars indicating measurements after 12 h (*t* 12) treatment. Data represent means ± SEM of four independent experiments performed in quadruplicate. Statistical analysis: One‐way ANOVA followed by Bonferroni's post hoc test. *p < 0.05, **p < 0.001, ***p < 0.0001 vs. vehicle; #*p* < 0.05, ##*p* < 0.01, ###*p* < 0.0001 vs. LPS; †*p* < 0.01, ††*p* < 0.001 vs. DSS.

To evaluate how MRME influences intestinal barrier function, we employed fully differentiated Caco‐2 cell monolayers. Exposure to inflammatory stimuli, specifically LPS (0.1 μg/mL for 12 h) or DSS (1% for 12 h), significantly reduced occludin levels, confirming a marked disruption of epithelial integrity (Figure 1). Notably, MRME pretreatment prevented this reduction in a dose‐dependent manner; the 10 μg/mL concentration proved most effective, significantly restoring occludin levels to those of vehicle‐treated controls (Figure 1).

Since MRME did not affect basal occludin levels, its protective mechanism appears to be specifically toward preventing external damage rather than modifying the physiological state. This protective activity was confirmed by occludin immunofluorescence analysis showing that MRME prevented the diffuse and fragmented patterns induced by LPS/DSS.

### 3.2 MRME Attenuates Oxidative Stress in the Co‐Cultured Neural Environment

**FIGURE 2:**
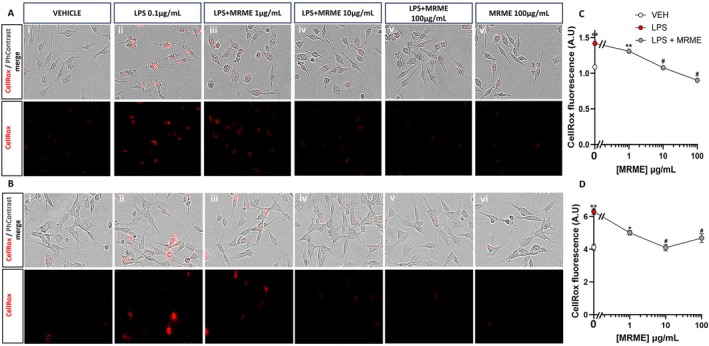
MRME prevents LPS‐induced oxidative stress in microglial and neuronal cells. Representative IncuCyte live‐cell imaging scans showing ROS production in BV2 microglia (A) and SH‐SY5Y neurons (B) under the following conditions: (i) vehicle (VEH), (ii) LPS (0.1 μg/mL), (iii) MRME (1 μg/mL) + LPS, (iv) MRME (10 μg/mL) + LPS, (v) MRME (100 μg/mL) + LPS, and (vi) MRME alone (100 μg/mL). Where indicated, cells were preincubated for 24 h with the increasing concentrations of MRME, followed by a 12 h treatment with LPS (0.1 μg/mL). Quantitative analysis of CellROX fluorescence intensity in BV2 (C) and SH‐SY5Y (D). Data are expressed as means ± SEM of four independent experiments, each performed in quadruplicate. Statistical analysis: One‐way ANOVA followed by Bonferroni's post hoc test (*p < 0.05, **p < 0.001 vs. vehicle; #*p* < 0.001 vs. LPS).

After establishing that MRME preserves the intestinal barrier physical integrity, we evaluated whether this protective effect translates into reduced oxidative stress in the basolateral “brain‐like” compartment (Figure 2). In our gut–brain co‐culture model, apical exposure to either LPS (0.1 μg/mL) or DSS (1%) triggered a significant increase in ROS production monitored via CellROX Deep Red in both BV2 microglia and SH‐SY5Y neurons (Figure 2A,B).

It is important to note that apical MRME pretreatment (1–100 μg/mL) dose‐dependently mitigated ROS production in the basolateral compartment. As shown by quantitative analysis and IncuCyte live cell scans (Figure 2C,D), the protective effect was most evident at 100 μg/mL. These results suggest that MRME prevents the transepithelial translocation of pro‐oxidant mediators, protecting neural cells from systemic inflammation.

### 3.3 MRME Mitigates DSS‐Induced Oxidative Stress in the Co‐Culture Model

**FIGURE 3:**
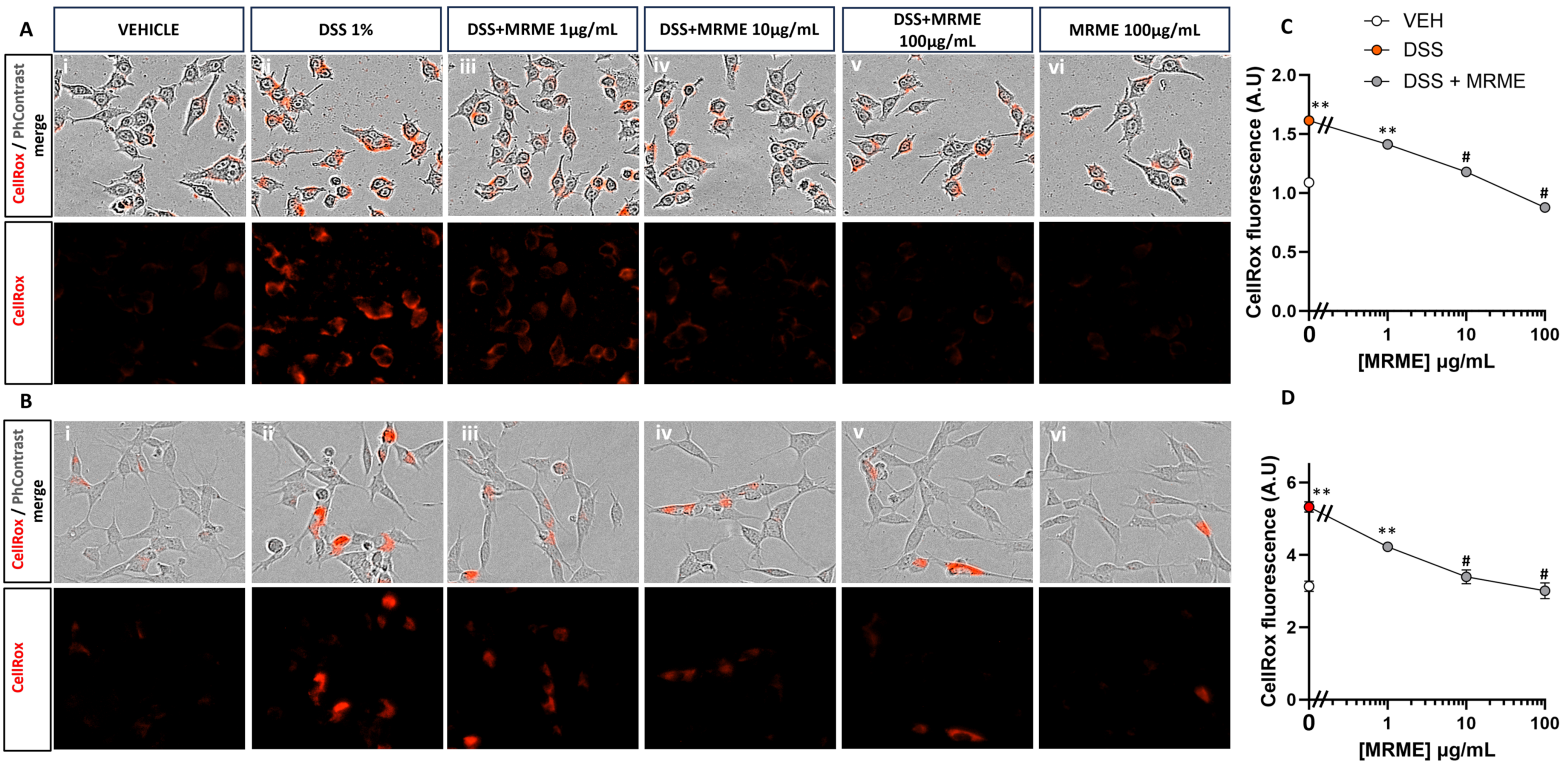
MRME attenuates DSS‐induced oxidative stress in microglial and neuronal cells. Representative IncuCyte live‐cell imaging scans showing ROS production in BV2 microglia (A) and SH‐SY5Y neurons (B) under the following conditions: (i) vehicle (VEH), (ii) DSS (1% for 12 h), (iii) MRME (1 μg/mL) + DSS, (iv) MRME (10 μg/mL) + DSS, (v) MRME (100 μg/mL) + DSS, and (vi) MRME alone (100 μg/mL). Where indicated, the cells were preincubated for 24 h with the increasing concentrations of MRME, followed by a 12 h treatment with DSS (1%). Quantitative analysis of CellROX fluorescence intensity in BV2 (C) and SH‐SY5Y (D) cells. Data are expressed as mean ± SEM of four independent experiments, each performed in quadruplicate. Statistical analysis: One‐way ANOVA followed by Bonferroni's post hoc test (*p < 0.05, **p < 0.001 vs. vehicle; #*p* < 0.001 vs. DSS).

Following the same experimental approach used for LPS, we evaluated whether MRME could protect the neural environment against a chemically‐induced intestinal insult (Figure 3). Apical administration of DSS (1%) to the differentiated Caco‐2 barrier resulted in a robust increase in ROS production in the basolateral compartment, as evidenced by CellROX Deep Red staining in both BV2 microglial and SH‐SY5Y neuronal cells (Figure 3A,B).

These results confirm that DSS‐induced epithelial damage triggers oxidative stress in the co‐cultured neural population. This effect was mitigated by apical MRME pretreatment (1–100 μg/mL) in a dose‐dependent manner. Quantitative analysis (Figure 3C,D) demonstrated that the 100 μg/mL concentration provided maximal protection, reducing ROS levels to near baseline values.

### 3.4 MRME Mitigates LPS‐Induced Apoptotic Cell Death in the Neural Compartment

**FIGURE 4:**
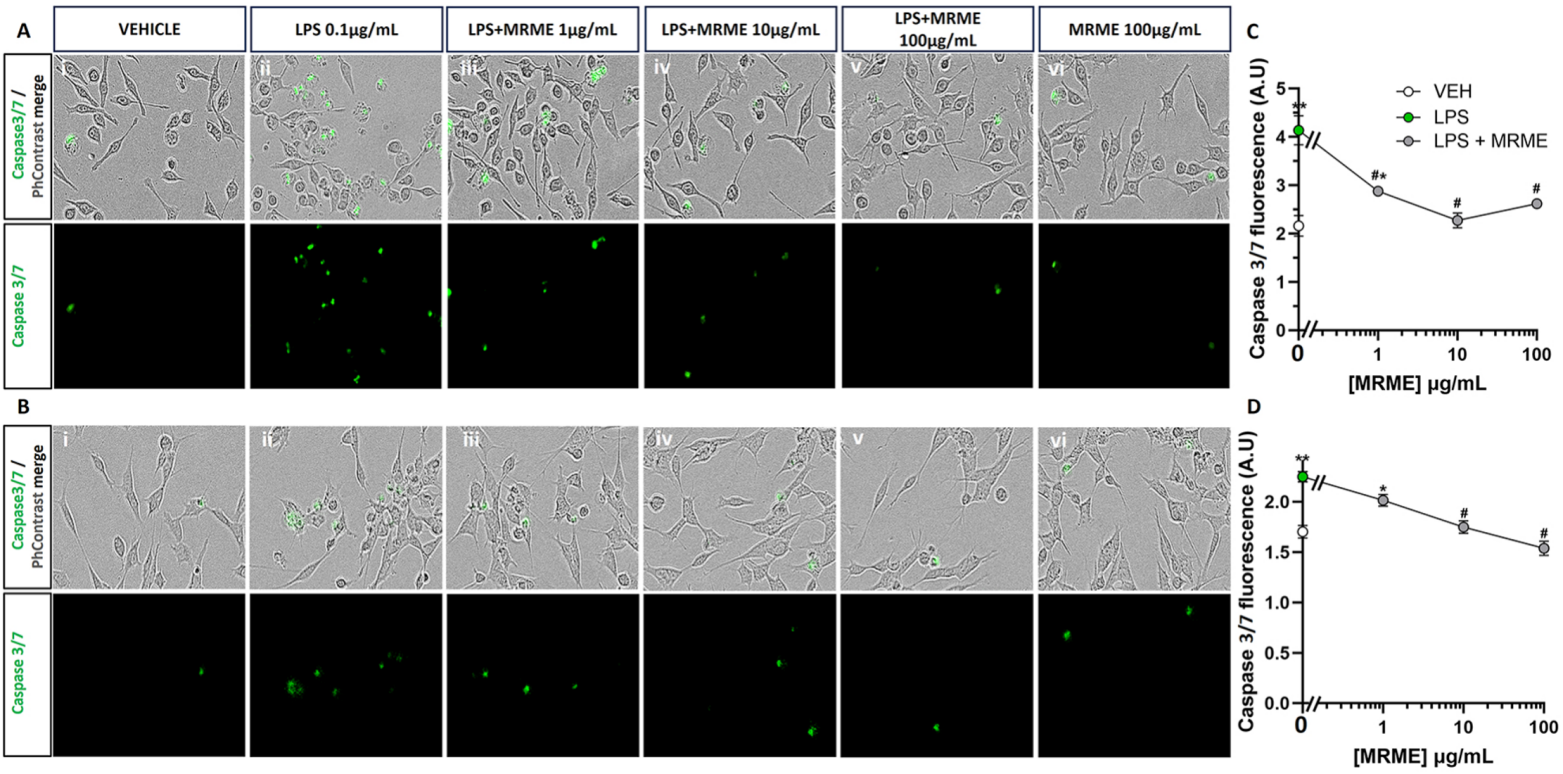
MRME mitigates LPS‐induced apoptosis in microglial and neuronal cells. Representative IncuCyte live‐cell imaging scans showing caspase‐3/7 activation in BV2 microglia (A) and SH‐SY5Y neurons (B) under the following conditions: (i) vehicle (VEH), (ii) LPS (0.1 μg/mL), (iii) MRME (1 μg/mL) + LPS, (iv) MRME (10 μg/mL) + LPS, (v) MRME (100 μg/mL) + LPS, and (vi) MRME alone (100 μg/mL). Where indicated, the cells were preincubated with the increasing concentrations of MRME (24 h), followed by a 12 h treatment with LPS (0.1 μg/mL). Quantitative analysis of caspase‐3/7 fluorescence intensity in BV2 (C) and SH‐SY5Y (D) cells. Data are expressed as mean ± SEM of four independent experiments, each performed in quadruplicate. Statistical analysis: One‐way ANOVA followed by Bonferroni's post hoc test (*p < 0.05, **p < 0.001 vs. vehicle; #*p* < 0.001 vs. LPS).

To further investigate the neuroprotective potential of the extract, we assessed the activation of caspase‐3/7 as a primary marker of apoptotic cell death (Figure 4). In our co‐culture system, apical stimulation with LPS (0.1 μg/mL) for 12 h induced a significant and sharp increase in caspase‐3/7 fluorescence intensity within the basolateral compartment, affecting both BV2 microglial and SH‐SY5Y neuronal populations (Figure 4A,B).

This increase in green fluorescence, monitored via live‐cell imaging, indicates that the inflammatory stress originating in the intestinal layer effectively triggers downstream apoptotic pathways in the neural environment. Preincubation with MRME (1, 10, and 100 μg/mL) on the apical side markedly reduced caspase‐3/7 activation in a concentration‐dependent manner. Quantitative analysis revealed that the highest dose of MRME (100 μg/mL) was particularly effective, significantly decreasing the apoptotic signal compared to cells treated with LPS alone (Figure 4C,D). Notably, MRME administered alone (100 μg/mL) showed no proapoptotic effects, maintaining fluorescence levels comparable to the vehicle control.

These results demonstrate that MRME confers neuroprotection by attenuating the cell death mechanisms driven by gut‐derived inflammatory insults.

### 3.5 MRME Mitigates DSS‐Induced Apoptotic Cell Death in the Neural Compartment

**FIGURE 5:**
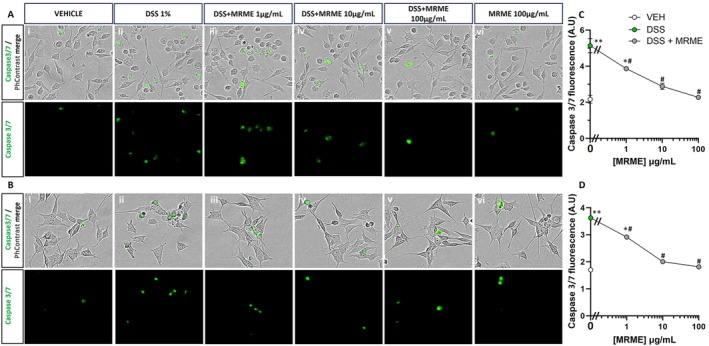
MRME mitigates DSS‐induced apoptosis in microglial and neuronal cells. Representative IncuCyte live‐cell imaging scans showing caspase‐3/7 activation in BV2 microglia (A) and SH‐SY5Y neurons (B) under the following conditions: (i) vehicle (VEH), (ii) DSS (1%, 12 h), (iii) MRME (1 μg/mL) + DSS, (iv) MRME (10 μg/mL) + DSS, (v) MRME (100 μg/mL) + DSS, and (vi) MRME alone (100 μg/mL). Where indicated, the cells were preincubated with the increasing concentrations of MRME (24 h), followed by a 12 h treatment with DSS (1%). Quantitative analysis of caspase‐3/7 fluorescence intensity in BV2 (C) and SH‐SY5Y (D) cells. Data are expressed as mean ± SEM of four independent experiments, each performed in quadruplicate. Statistical analysis: One‐way ANOVA followed by Bonferroni's post hoc test (*p < 0.05, **p < 0.001 vs. vehicle; #*p* < 0.001 vs. DSS).

Similarly to the effects observed with LPS, we evaluated whether MRME could prevent apoptotic cell death induced by apical DSS‐mediated stress (Figure 5). The exposure of the Caco‐2 barrier to DSS (1%) significantly triggered the activation of caspase‐3/7 in both BV2 microglia and SH‐SY5Y neurons in the basolateral compartment (Figure 5A,B).

The increase in green fluorescence intensity confirms that chemical disruption of the intestinal epithelial layer leads to a proapoptotic response in the underlying neural cells.

Apical MRME pretreatment (1–100 μg/mL) reduced in a dose‐dependent manner caspase‐3/7 activation in the basolateral compartment. As demonstrated by quantitative analysis (Figure 5C,D), the apoptotic signal was significantly attenuated at the highest concentrations of the extract (100 μg/mL), protecting the neural compartment from DSS‐mediated damage.

### 3.6 Effect of MRME on Pathogenic Bacterial Proliferation

**FIGURE 6:**
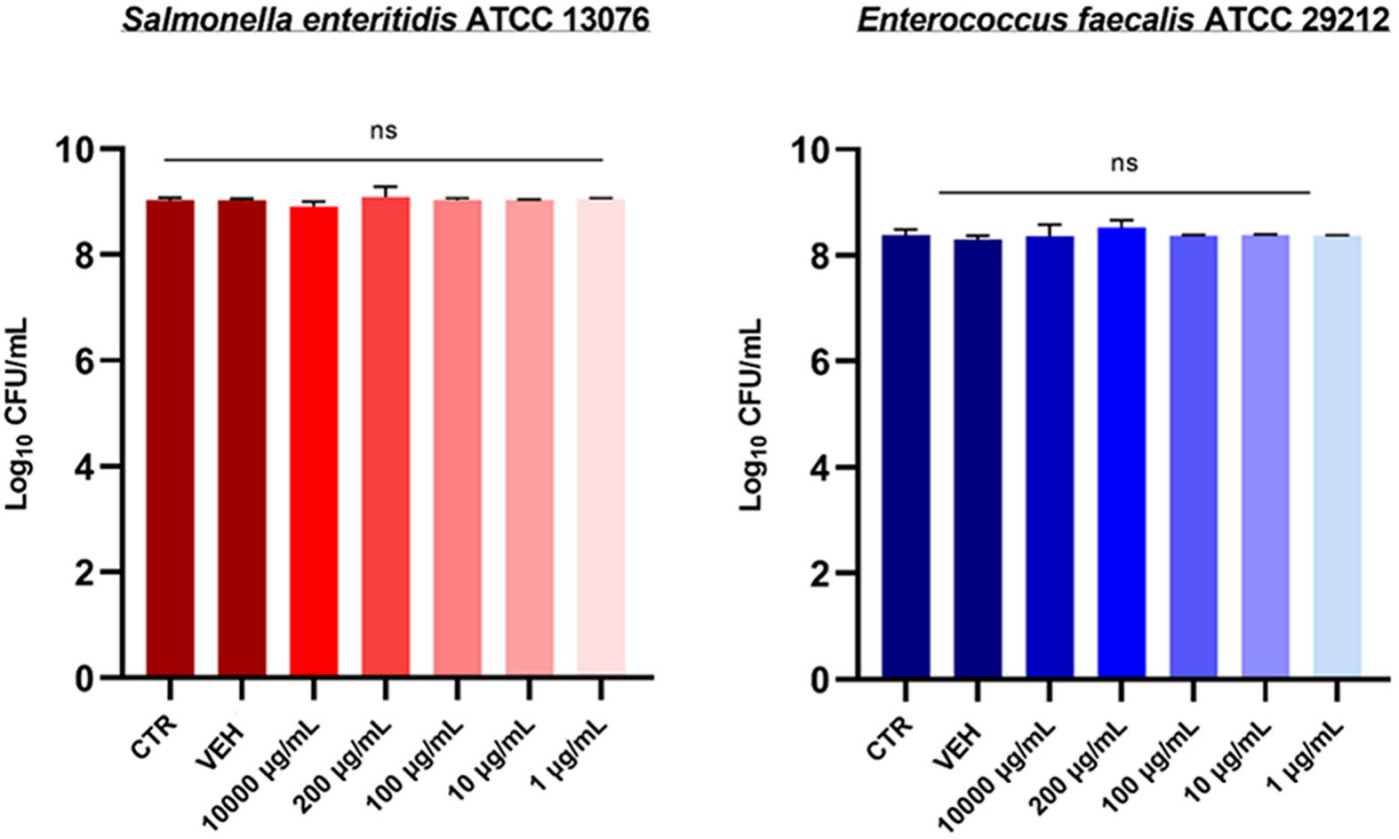
Effect of MRME on the pathogenic bacterial strains viability. *Salmonella enteritidis* ATCC13076 and *Enterococcus faecalis* ATCC29212 were grown in the presence of increasing concentrations of MRME (1, 10, 100, 200, and 10,000 μg/mL). Data are expressed as the mean ± SD of log_10_ CFU/mL from three independent experiments. Statistical analysis: Two‐way ANOVA (no significant differences were observed; *p* > 0.05 vs. controls).

To investigate its potential antimicrobial properties or direct toxicity toward the microbiota, MRME was tested against representative Gram‐negative ( *Salmonella enteritidis* ATCC 13076) and Gram‐positive ( *Enterococcus faecalis* ATCC 29212) pathogen strains. The extract was evaluated over a broad concentration range, from 1 to 10,000 μg/mL. MRME exhibited no bactericidal activity against either strain (Figure 6).

Increasing concentrations of MRME did not alter bacterial proliferation compared to the control, indicating that the MRME protective effects were independent of direct antibacterial activity but are instead attributable to the modulation of host‐specific cellular pathways.

### 3.7 Safety Profile of MRME on Probiotic Bacterial Strains

**FIGURE 7:**
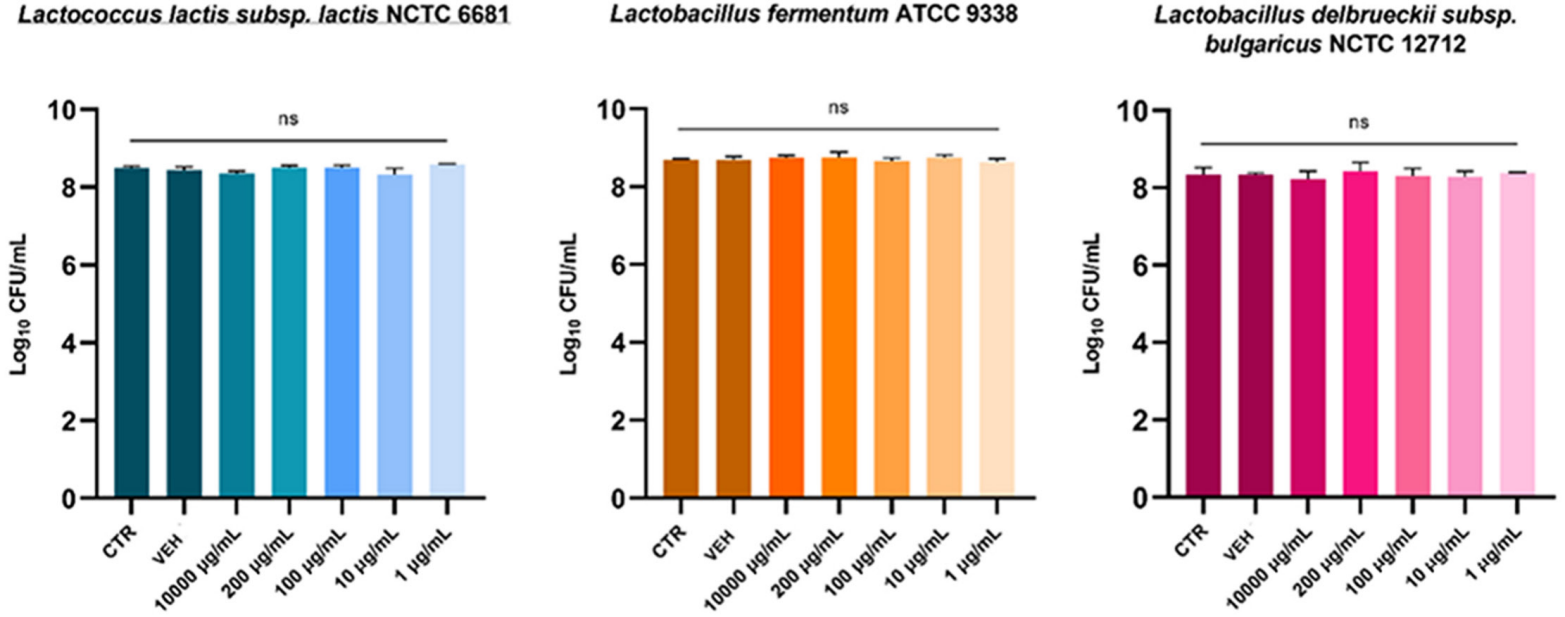
Effect of MRME on the viability of beneficial probiotic strains. *Lactococcus lactis* subsp. *lactis* NCTC 6681, *Lactobacillus fermentum* ATCC 9338, and *Lactobacillus delbrueckii* subsp. *bulgaricus* NCTC 12712 were grown in the presence of increasing concentrations of MRME (1, 10, 100, 200, and 10,000 μg/mL). Data are expressed as the mean ± SD of log_10_ CFU/mL from three independent experiments. Statistical analysis: Two‐way ANOVA (no significant differences were observed; *p* > 0.05 vs. controls).

To test whether the effects of MRME negatively impact the beneficial gut microbiota, we evaluated its influence on the growth of three representative probiotic strains: *Lactococcus lactis* subsp. *lactis* NCTC 6681, *Lactobacillus fermentum* ATCC 9338, and *Lactobacillus delbrueckii* subsp. *bulgaricus* NCTC 12712. MRME demonstrated an excellent safety profile. No inhibitory or bactericidal effects were observed at any of the tested concentrations, ranging from 1 to 10,000 μg/mL (Figure 7).

The bacterial density, measured as log_10_ CFU/mL, remained stable and comparable to both the untreated control (CTR) and the vehicle (VEH) for all three species. Statistical analysis (*p* > 0.05) confirmed that, at the doses tested, MRME does not interfere with the proliferation of these beneficial bacteria, supporting its potential as a safe pharmacological agent that preserves the ecological balance of the intestinal microbiota while exerting its protective role on the epithelial barrier.

## 4 Discussion

This study provides compelling evidence that Mela Rosa Marchigiana pulp callus extract acts as a potent multitarget agent within the gut–brain axis. By employing a well‐established and validated transwell co‐culture system, we simulated the physiological communication between a fully differentiated intestinal epithelium (Caco‐2) and the underlying neuro‐microglial niche (SH‐SY5Y and BV2 cells). Our results demonstrate that MRME stabilizes the intestinal barrier, a mechanism that finds a strong parallel in the literature regarding pentacyclic triterpenes. The ability of MRME to preserve TEER and occludin integrity (Figure 1) during LPS or DSS induced oxidative stress, emphasized its protective potential. This supports earlier findings that triterpenic acids, such as tormentic acid can enhance epithelial integrity through the modulation of protein kinase pathways (e.g., PKC/Rho kinase) and stabilizing TJ proteins [26]. By stabilizing the intestinal barrier, MRME neutralizes the “leaky gut” and contain the initial insult before systemic damage can occur. One of the most significant potential consequences of this effect is the neuroprotective actions observed in the basolateral environment. Despite apical administration, we observed a robust, dose‐dependent reduction in ROS production (Figures 2 and 3) and caspase‐3/7‐mediated apoptosis (Figures 4 and 5) in both neurons and microglia. Indeed, while active metabolites in MRME may cross the Caco‐2 monolayer, the apical pretreatment protocol strongly suggests a barrier‐mediated effect on the basolateral cellular components. Thus, MRME may work through “signal transduction” across the epithelium. This dual mechanism, which provides for physical shielding and modulation of the epithelial secretome, is similar to that shown by other polyphenolic and triterpenic complexes. For instance, triterpenoids from *Alnus japonica* have been shown to suppress IL‐6 and IL‐8 secretion in intestinal epithelial cells [40], while broader terpene complexes reinforce barrier integrity and inhibit the TNF‐α/NF‐κB signaling axis [41, 42]. The MRME‐mediated attenuation of the “oxidative burst” (Figures 2C and 3C), indicates the maintenance of a quiescent microglial state, inhibiting proinflammatory polarization [43]. These findings are particularly relevant since chronic microglial activation is a hallmark of neurodegeneration. Consistently, the reduced caspase activation in SH‐SY5Y neurons (Figures 4D and 5D) confirms the ability of MRME to preserve neuronal integrity, aligning the neuroprotective effects observed with those of other dietary triterpenoids [44, 45].

Future studies will need to address the question of whether the effects of MRME on ROS production and apoptosis of microglial and neuronal cells are indeed entirely mediated by its effects on the intestinal epithelial cell barrier and accompanied by mitigation of proinflammatory cytokine release by these cells.

Bioactive extracts often risk triggering dysbiosis through nonspecific antimicrobial effects; consequently, gut microbiota compatibility is essential. Our results (Figures 6 and 7) confirm that MRME shows a distinct “ecological neutrality” with respect to strains tested. This “microbiota safe” profile suggests that its neuroprotective benefits are achieved without disrupting the microbial composition or the functional niches essential to produce neuroactive metabolites.

While the literature often reports antimicrobial properties for isolated triterpenoids, our data align with that reported by Rodriguez‐Daza et al. [46] who emphasize how therapeutic extracts should target host tissues without triggering dysbiosis. Notably, Kurek et al. [47] demonstrated that isolated oleanolic and ursolic acids exert bactericidal effects on *Listeria monocytogenes* . Similarly, Kim et al. [48] reported significant bacterial inactivation (3–4 log_10_ CFU/mL) not only for *Listeria monocytogenes* but also for *Enterococcus faecium* and *Enterococcus faecalis* when treated with pure oleanolic acid. The lack of bactericidal effects of MRME evidenced here highlights the “phytocomplex advantage” in MRME, where the high concentrations of tormentic (40.2%) and annurcoic (16.6%) acids may act synergistically with other matrix components to prioritize host‐cell modulation over direct bacterial toxicity. Unlike purified compounds that can disrupt microbial membranes, the complex signature of MRME appears to support microbial homeostasis.

## 5 Conclusion

Our findings provide strong evidence that MRME acts as a comprehensive modulator of the gut–brain axis through a sophisticated multilevel mechanism. By reinforcing the intestinal barrier and preserving TJ integrity, thus counteracting the translocation of systemic stressors, MRME may exert the other effects observed here, i.e., modulation of ROS production and neural cell apoptosis, with potential mitigation of downstream neuroinflammation and neuronal damage. Notably, these benefits would be achieved with a remarkable degree of “ecological neutrality” since, unlike many broad‐spectrum bioactive compounds, MRME does not interfere with the growth of essential commensal and psychobiotic strains, such as *Lactobacillus fermentum* , potentially ensuring the maintenance of a functional microbial niche for neuroactive metabolite production.

Our results highlight the potential of MRME as a safe and noninvasive therapeutic candidate for disorders characterized by gut–brain axis dysregulation. Given its ability to preserve homeostasis without triggering dysbiosis, MRME could represent a novel adjuvant strategy in the prevention or management of neurodegenerative conditions related to the “leaky gut” and ensuing chronic neuroinflammation. Future studies are warranted to validate these protective effects in vivo and to explore the long‐term benefits of MRME's unique triterpenic profile. In this context, the high concentrations of tormentic and annurcoic acids in MRME provide a standardized phytochemical fingerprint, which is essential for the development of reproducible and reliable nutraceutical formulations.

## Author Contributions

Conceptualization: Maria Cristina Albertini and Letizia Palomba. Data curation: Alessandro Nicois, Daniele Fraternale, Brenda Marfella, Mariastella Colomba, and Barbara Di Giacomo. Formal analysis: Alessandro Nicois, Daniele Fraternale, Francesco Palma, and Giuditta Fiorella Schiavano. Funding acquisition and co‐funding: Letizia Palomba and Vincenzo Di Marzo. Investigation: Alessandro Nicois, Daniele Fraternale, Luigia Cristino, and Maria Cristina Albertini. Methodology: Alessandro Nicois, Daniele Fraternale, Brenda Marfella, Francesco Palma, and Giuditta Fiorella Schiavano. Project administration and resources: Letizia Palomba. Supervision: Letizia Palomba. Visualization: Mariastella Colomba and Barbara Di Giacomo. Writing – original draft: Alessandro Nicois, Daniele Fraternale, Vincenzo Di Marzo, and Letizia Palomba. Writing – review and editing: Brenda Marfella, Francesco Palma, Giuditta Fiorella Schiavano, Mariastella Colomba, Armando Gregorini, Barbara Di Giacomo, Luigia Cristino, Vincenzo Di Marzo, and Maria Cristina Albertini.

## Funding

This research was supported by the Department of Biomolecular Sciences, University of Urbino Carlo Bo [project code: DISB_PALOMBA_PROG24].

## Conflicts of Interest

The authors declare no conflicts of interest.

## Data Availability

The data that support the findings of this study are available from the corresponding author upon reasonable request.
